# Phenotypic Characterization of Intellectual Disability Caused by *MBOAT7* Mutation in Two Consanguineous Pakistani Families

**DOI:** 10.3389/fped.2020.585053

**Published:** 2020-12-01

**Authors:** Liwei Sun, Amjad Khan, Han Zhang, Shirui Han, Xiaerbati Habulieti, Rongrong Wang, Xue Zhang

**Affiliations:** State Key Laboratory of Medical Molecular Biology, McKusick-Zhang Center for Genetic Medicine, School of Basic Medicine Peking Union Medical College, Institute of Basic Medical Sciences Chinese Academy of Medical Sciences, Beijing, China

**Keywords:** in-frame deletion, Pakistani consanguineous families, intellectual disability, founder effect, *MBOAT7* gene

## Abstract

A homozygous in-frame deletion (c. 758_778del; p. Glu253_Ala259del) in membrane-bound O-acyltransferase family member 7 (*MBOAT7)*, also known as lysophosphatidylinositol acyltransferase (LPIAT1), was previously reported to be the genetic cause of intellectual disability (ID) in consanguineous families from Pakistan. Here, we identified two additional Pakistani consanguineous families with severe ID individuals sharing the same homozygous variant. Thus, we provide further evidence to support this *MBOAT7* mutation as a potential founder variant. To understand the genotype-phenotype relationships of the in-frame deletion in the *MBOAT7* gene, we located the variant in the fifth transmembrane domain of the protein and determined that it causes steric hindrance to the formation of an α-helix and hydrogen bond, possibly influencing its effectiveness as a functional transmembrane protein. Moreover, extensive neuropsychological observations, clinical interviews and genetic analysis were performed on 6 patients from the 2 families. We characterized the phenotype of the patients and noted the serious outcome of severe paraplegia. Thus, optimal management for symptom alleviation and appropriate screening in these patients are crucial.

## Introduction

Intellectual disability (ID) is a neurodevelopmental disorder characterized by substantial limitations in intellectual functioning and adaptive behavior (1). ID has an estimated prevalence of 1–3% worldwide and an increased prevalence among inbred consanguineous populations (2). ID shows extreme clinical and genetic heterogeneity. It can occur in isolation or in combination with congenital malformations or other neurological features, such as epilepsy, sensory impairment and autism spectrum disorders (ASD), and its severity is highly variable, ranging from mild to severe. The genetics of ID are heterogenetic with variable causes, including chromosomal aberrations (3, 4), copy number variations (CNVs) (5, 6), autosomal dominant (7), autosomal recessive (8, 9), and X-linked variants (10). *De novo* mutation is a major cause of sporadic ID cases among outbred populations, while autosomal recessive intellectual disability (ARID) is the leading inheritance form of ID in countries with frequent parental consanguinity (11, 12). Since membrane-bound O-acyltransferase family member 7 (*MBOAT7*, OMIM 606048) was reported as the causative gene for ID in six consanguineous ID families from Pakistan for the first time (13), to date, 16 mutations in the *MBOAT7* from a total of 21 families have been observed, including 7 frameshift mutations (13–17), 2 splice mutations (13, 18), 3 non-sense mutations (16, 17), 2 missense mutations (12, 16), a 21-bp deletion (13), an 11,594bp deletion (16), and an indel variant (19). Heterogeneous clinical features were associated with *MBOAT7* defect, such as moderate to severe intellectual disability, epilepsy, developmental delay, attention-deficit hyperactivity disorder (ADHD), microcephaly or macrocephaly, and autistic features. Here, we delineated the detailed clinical and genetic data of individuals with severe ID from two consanguineous Pakistan families harboring the same in-frame deletion in *MBOAT7*. We also performed extensive neuropsychological observations and clinical interviews on all 6 affected individuals from families. We found more complex psychiatric dysfunction or behavioral problems, such as feeding refusal, self-injurious behavior and severe paraplegia, in the probands in addition to features reported previously, broadening the clinical manifestation spectrum.

## Materials and Methods

### Ethics and Consent Statement

Clinical information and blood samples were collected from the probands and available family members, and informed consent was obtained. Genetic testing was performed in accordance with the Helsinki Declaration and was approved by the Peking Union Medical College Institutional Review Board.

### Patients and DNA Extraction

We recruited six patients with severe ID from two consanguineous families from the Khyber Pakhtunkhwa Province of Pakistan. Peripheral blood samples (3–5 ml) were collected for genomic DNA extraction using the QIAamp DNA Blood Midi Kit (Qiagen, Hilden, Germany) and quantified using a Nanodrop 2000 spectrophotometer (Thermo Scientific, Waltham, MA, United States).

### Whole-Exome Sequencing

Whole-exome sequencing (WES) was performed to detect variants in the proband and available family members (Family 1: II-2, III-4, IV-4, IV-5; Family 2: III-5, III-6, IV-9, IV-12). Sequencing libraries were generated using the Agilent SureSelect Human All Exon V6 kit (Agilent Technologies, CA, USA). DNA libraries were sequenced using an Illumina HiSeq platform with a 100 × read depth. Valid sequencing data were mapped to the reference human genome (UCSC hg19) using Burrows–Wheeler Aligner (BWA) software. Samtools (20) mpileup and bcftools were used to perform variant calling and identify SNPs, insertions and deletions (InDels). ANNOVAR (21) was performed to annotate in VCF (Variant Call Format). Functional annotation was performed based on the databases such as dbSNP, 1000 Genome, Consensus CDS, RefSeq, Ensembl and UCSC.

### Mutation Analysis

Variants were filtered based on the following criteria: [1] occurring in coding regions and/or splice sites; [2] non-synonymous; [3] found at < 0.1% frequency (Single Nucleotide Polymorphism database [dbSNP] and Exome Variant Server); and [4] homozygous in consanguineous families. The variants were further confirmed by PCR amplification using specific primers (forward primer: 5′-CTGCTGGGTCTTGGGAAG-3′; reverse primer: 5′-TGCTGTTCCTGCTCTCCTCT-3′). Sanger sequencing was performed for further validation using standard protocols. The PCR products were sequenced using ABI BigDye3.1 (Applied Biosystems, Foster City, CA, USA) and were analyzed using the ABI 3730XL sequencer.

### Bioinformatic Pathogenicity Predictions

The pathogenicity for the in-frame deletion was predicted by the *in silico* prediction tools PROVEAN (http://provean.jcvi.org/index.php), Mutation Taster (http://www.Mutationtaster.org/), and MutPred-Indel (http://mutpred2.mutdb.org/cgi-bin/mutpred2_output.py).

### Molecular Modeling and Structural Analysis

The 3D modeled structures of the MBOAT7 proteins for the wild-type and mutant types were prepared using homology modeling in SWISS-MODEL (https://swissmodel.expasy.org/). Structural analysis and attribution of the residue interaction networks to the protein function were analyzed and visualized by PyMOL software (https://pymol.org/2/).

## Results

### Clinical Characterization

#### Family 1

**Figure 1:**
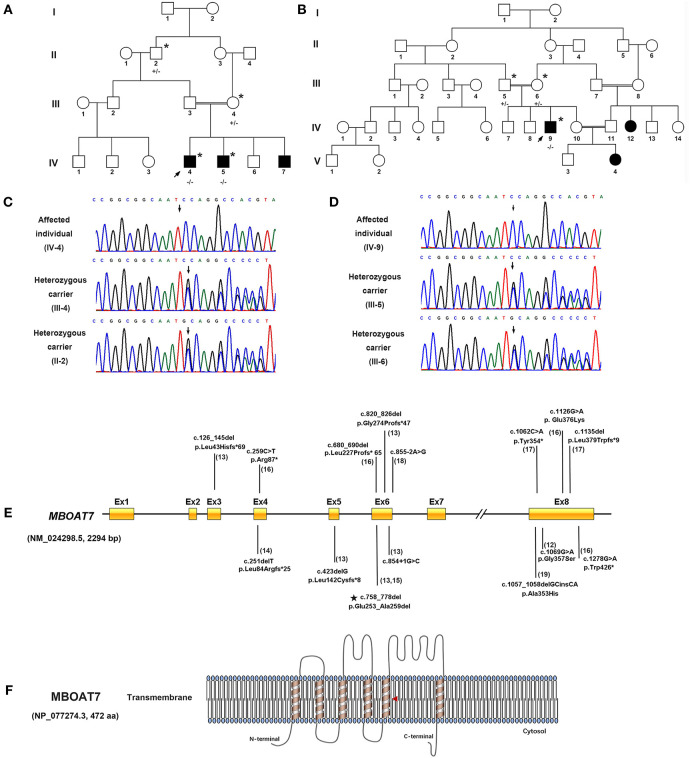
Identification of a homozygous in-frame deletion in *MBOAT7* in individuals with severe intellectual disability in Pakistani consanguineous families. **(A)** Pedigrees of consanguineous families of Family 1. **(B)** Pedigrees of consanguineous families of Family 2. A double bar represents parental consanguinity. Male subjects are represented by squares, female subjects by circles and affected individuals by shading; the arrow indicates the proband. Individuals who had undergone genetic testing are marked with an asterisk (+/+, wild type; +/–, heterozygous carrier; –/–, homozygous in-frame deletion). **(C,D)** Electropherogram showing the DNA sequence of the in-frame deletion variant (c.758_778del [p. Gln253_Ala259del]) in *MBOAT7* (NM_024298.5, 2294 bp). **(E)** Genetic structure of *MBOAT7*. Mutations are indicated by arrows, and the in-frame deletion variant detected in the present work is indicated by a pentagram. **(F)** Location of the variant within the transmembrane domain of MBOAT7 protein (NP_077274.3, 472 amino acids) as shown in the red triangle (aa, amino acids).

In family 1 (Figure 1A), the proband (IV-4) is an 11-year-old boy who had severe intellectual disability. When he was 2–4 years old, he presented with early-onset febrile epilepsy, severe speech and language communication disorders, and developmental delay. He also had symptoms of feeding refusal. Remarkably, he presented with self-injurious behavior, banging of the head against the wall or hand, screaming at home, particularly during epileptic attacks, and aggressive behavior. Enuresis and somniloquy were observed. He had dysmorphic features, including large and low set ears, a depressed nasal bridge, strabismus, epicanthus, a flat philtrum and relatively thin upper lips with mild wide spacing of teeth. At the last examination (11 years of age), his weight was 45.9 kg (+ 1.1 SD), his height was 121.6 cm (−3.4 SD), and the OFC was 52.1 cm (−0.9 SD). Karyotype analysis was normal (46 XY). Other clinical features, such as hearing, cardiac, respiratory, nose, nails and skin, appeared normal. The proband also had two brothers (IV-5 and IV-7) who presented with the same symptoms, and all these individuals presented the disease features when they were 2–4 years old.

#### Family 2

In Family 2 (Figure 1B), the proband (IV: 9) is a 20-year-old young man. He walked at 18 months of age and showed gross and fine motor incoordination. He presented with severe speech delay and non-verbal learning disorders, social impairment, cognitive disorders, and developmental delays. He was found to have severe early-onset epilepsy. At the last examination (20 years of age), he measured 169.7 cm in height, weighed 65.7 kg and had an OFC (occipital circumference) of 54 cm (−0.3SD). Importantly, he had severe self-injurious behavior and ADHD. Dysmorphic features include prominent eyes with mild hypertelorism and downslanting palpebral fissures. In addition to the initial motor incoordination during infancy, our clinical follow-up also demonstrated that the symptom had developed into severe paraplegia, with his walking ability lost completely and becoming bedridden. Two affected females (IV: 12 and V: 4) in the family also presented with speech delays, ID, DD, seizures, ADHD, and mild social impairment. The clinical manifestations of all six severe ID individuals from the two families are summarized in Table 1.

**Table 1:** Summary of the clinical features of the present study and published cases associated with *MBOAT7* variants.

	**Present Study**						**Khan et al. (15)**	**Johansen et al. (13)**	**Hu et al. (12)**	**Santos-Cortez et al. (14)**	**Yalnizoglu et al. (16)**	**Jacher et al. (18)**	**Heidari et al. (17)**	**Farnè et al. (19)**
**Number of Families**	**Family 1**			**Family 2**			**2**	**6**	**1**	**1**	**7**	**1**	**2**	**1**
Gender (number of individuals)	Male (IV-4)	Male (IV-5)	Male (IV-7)	Male (IV-9)	Female (IV-12)	Female (V-4)	Male (4); Female (3)	Male (7); Female (9)	Male (3); Female (0)	Male (2); Female (2)	Male (5); Female (7)	Male (0); Female (1)	Male (2); Female (1)	Male (0); Female (1)
Origin	Pakistan	Pakistan	Pakistan	Pakistan	Pakistan	Pakistan	Pakistan	Egypt (3/16); Pakistan (9/16); Jordan (2/16); Iraq (2/16)	Iran (3/3)	Pakistan	Turkey (12/12)	NA	Iran (3/3)	Italy (1/1)
**Development** Intellectual disability	+	+	+	+	+	+	+	16/16	3/3	4/4	12/12	1/1	3/3	1/1
Developmental delay	+	+	+	+	+	+	+	16/16	0/3	NA	12/12	1/1	3/3	1/1
Speech delay/impairment	+	+	+	+	+	+	+	14/16	0/3	4/4	12/12	1/	A few words (3/3)	1/1
Severe paraplegia^*^	NA	NA	NA	+	NA	NA	NA	NA	NA	NA	NA	NA	NA	NA
**Neurological features** Seizures	+	+	+	+	+	+	+	16/16	3/3	4/4	11/12	1/1	3/3	1/1
ADHD	NA	NA	NA	+	+	+	NA	0/16	NA	NA	NA	1/1	NA	NA
**Behavior** Impairment of social interaction	+	+	+	+	+	+	+	16/16	NA	NA	12/12	1/1	3/3	1/1
Self-injurious behavior^*^	+	+	+	+	NA	NA	NA	NA	NA	NA	NA	NA	NA	NA
Feeding refusal^*^	+	+	+	NA	NA	NA	NA	NA	NA	NA	NA	NA	NA	NA
Hyperphagia/obesity	NA	NA	NA	NA	NA	NA	NA	NA	NA	NA	NA	1/1	NA	1/1
Autistic features	NA	NA	NA	NA	NA	NA	3/7	7/16	3/3	4/4	4/12	1/1	2/3	1/1
Dysmorphic features	Low set ears, depressed nasal bridge, strabismus, normal lips	NA	NA	Prominent eyes with mild hypertelorism; down slanting palpebral fissures	NA	NA	NA	NA	Strabismus	NA	Apathetic face, large ears, deep set eyes, short philtrum, and broad forehead	Nystagmus, strabismus with amblyopia	NA	Curly hair, heavy eyebrows, synophrys, high nasal bridge, full cheeks, and facial hirsutism

*NA, not applicable; ADHD, attention-deficit hyperactivity disorder*.

*Note: +, feature presented*.

* *indicates the expanding features observed in our study*.

### Genomic Sequencing and Analysis

**Figure 2:**
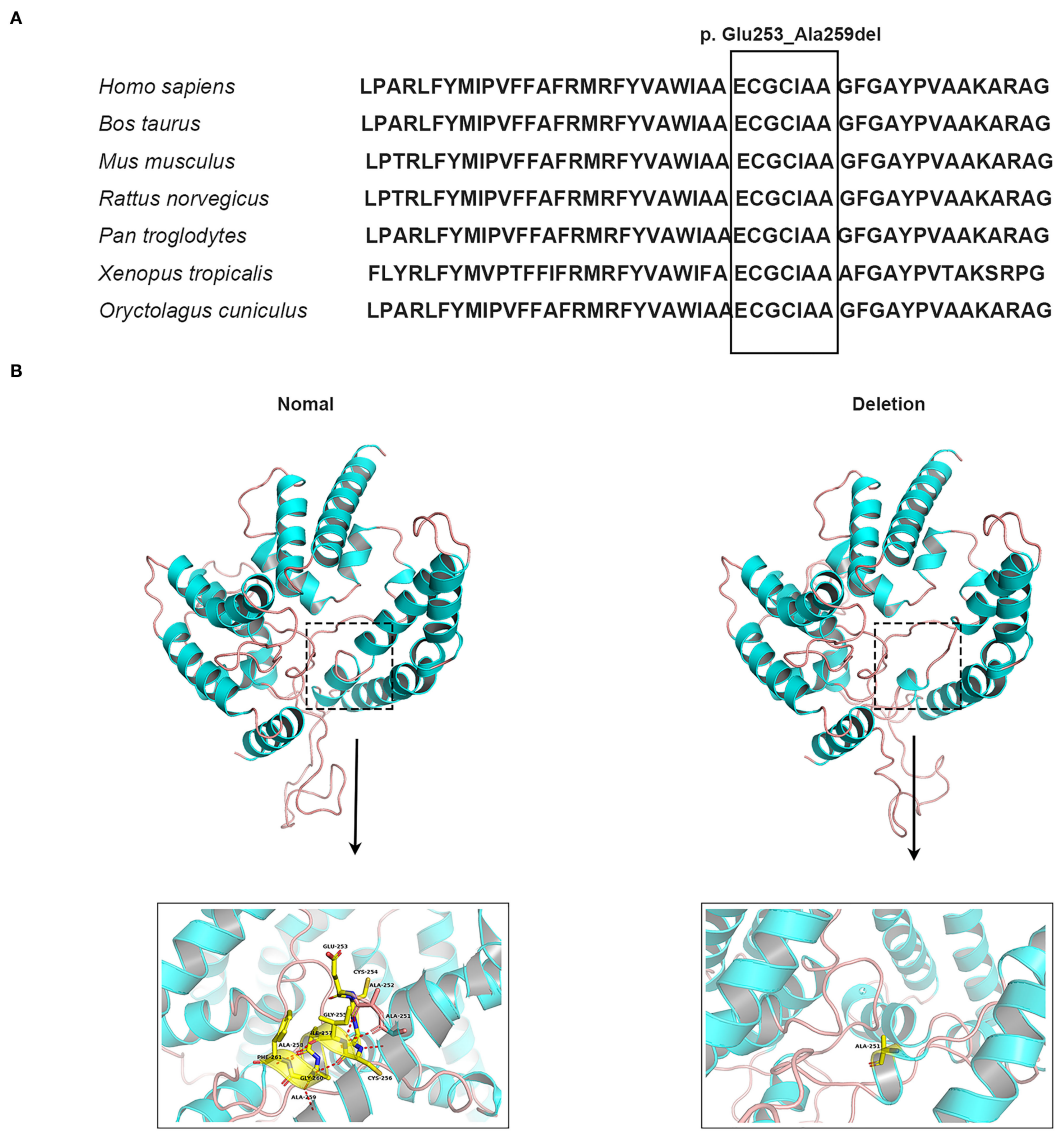
Conservation analysis and molecular modeling of wild-type and mutant MBOAT7 protein. **(A)** Conservation analysis of the in-frame deletion variant (c.758_778del [p. Gln253_Ala259del]) in the MBOAT7 protein. MBOAT7 protein sequence alignment from different organisms indicates that the in-frame deletion variant is highly conserved in mammalian and vertebrate species: *Homo sapiens* (NP_077274.3), *Bos taurus* (NP_001068620.1), *Mus musculus* (NP_084210.2), *Rattus norvegicus* (NP_001128450.1), *Pan troglodytes* (XP_009434639.1), *Xenopus tropicalis* (XP_012822891.2), *Oryctolagus cuniculus* (DP001062.1). **(B)** Three-dimensional schematic of the structure of normal MBOAT7 protein (on the left) and the MBOAT7 protein with an in-frame deletion (p. Gln253_Ala259del) (on the right). The alteration in the three-dimensional positioning of the in-frame deletion causes steric hindrance to the formation of helices and hydrogen bonds in the MBOAT7 protein. The structure highlighted by the dotted box can be viewed magnified as indicated by the arrow. The red dots show hydrogen bonds.

Whole-exome sequencing identified the same in-frame deletion in exon 6 (c.758_778del, p. Glu253_Ala259del) of *MBOAT7* (GenBank: NM_024298.5) in the three available affected individuals. The variant was confirmed by Sanger sequencing (Figures 1C,D), and it segregated with the disease as a fully penetrant recessive trait within families. *MBOAT7* harbored eight exons and four protein-coding transcripts, and the variants described until now affected all protein-coding transcripts, as shown (13, 16, 18) (Figure 1E). The *MBOAT7* variant (c. 758_778del; p. Glu253_Ala259del) has an allele frequency of 0.000032 within the global population and an allele frequency of 0.0001 within the Asian population in gnomAD (The Genome Aggregation Database, gnomAD) and an allele frequency of 0.000098 within the global population and an allele frequency of 0.00018 within the Asian population in ExAC (The Exome Aggregation Consortium, ExAC). It is located within highly conserved amino acid residues (Figure 2A). The variant is interpreted as pathogenic in Clinvar and dbSNP and is considered deleterious and disease causing by pathogenicity analysis using several *in silico* prediction tools, including PROVEAN, Mutation Taster, and MutPred-Indel.

### *In silico* Analysis and Protein Structure Modeling

Because *MBOAT7* is involved in the Lands' cycle, which is a metabolic pathway in the endoplasmic reticulum compartment, it influences the composition of membranes by remodeling the acyl chain composition of phospholipids by anchoring to endomembranes by six transmembrane domains (22). The variant detected in our study was located in the fifth transmembrane domain (Figure 1F). The wild-type and mutant MBOAT7 protein structures showed that the alteration in the three-dimensional positioning of the in-frame deletion caused steric hindrance to the formation of an α-helix and hydrogen bond, which may influence its transmembrane protein function (Figure 2B).

## Discussion

In this study, we identified a homozygous in-frame deletion (c.758_778del; p. Glu253_Ala259del) in *MBOAT7* causing severe intellectual disability in two consanguineous Pakistani families. The variant has been reported in Pakistani families previously, indicating it could be a regional prevalent founder mutation. We characterized the phenotypes in 6 subjects from the two families, all of whom presented with clinical signs during infancy or early childhood, including at 18 months old (3/6 subjects) and 2–4 years old (3/6 subjects). Severe ID accompanied by features of speech impairment, social interaction skills impairment, development delays and locomotor incoordination was observed in all of the subjects. Moreover, they all manifested early childhood-onset epilepsy, supporting epilepsy as a consistent feature of *MBOAT7*-related ID. Additionally, we characterized unique features, such as severe paraplegia (1/6 subjects), feeding refusal (3/6 subjects), and self-injurious behavior (4/6 subjects). In contrast to the feature of feeding refusal, hyperphagia and obesity were previously reported in 4 patients (12, 17). Notably, severe paraplegia was observed for the first time among the conditions of *MBOAT7* gene defects, possibly resulting from truncal hypotonia and progressive spasticity and indicating the importance of early detection, timely treatment and regular follow-up observations to reduce the risk of such serious outcomes.

*MBOAT7* is localized in the endoplasmic reticulum, mitochondria-associated membrane and lipid droplets, which play vital roles during the process of hepatic phospholipid remodeling and a non-canonical hepatic triglyceride synthesis pathway (23, 24). Increasing evidence indicates that the genetic variation of rs641738 in *MBOAT7* increases the susceptibility risk to liver disease. However, none of our patients manifested relevant liver disease, likely because the patients are still very young.

Therefore, we suggest that a full neurological psychiatric assessment should be performed on patients with *MBOAT7* gene defects, considering the variant in the presence of similar manifestations in children, particularly those from consanguineous families, so that it can help to benefit the early diagnosis and timely treatment for symptom alleviation and improvement of quality of life.

## Conclusion

Our findings further confirm the association of the potential founder homozygous in-frame deletion (c. 758_778del; p. Glu253_Ala259del) in the *MBOAT7* Gene and severe ID. The phenotypic characterization of ID in the two consanguineous Pakistani families expands the phenotypic spectrum of *MBOAT7*-related ID. Therefore, we suggest targeted screening for the variant in relevant clinical circumstances.

## Data Availability Statement

The datasets generated for this study can be found in online repositories. The names of the repository/repositories and accession number(s) can be found at: https://www.ncbi.nlm.nih.gov/genbank/, NM_024298.5.

## Ethics Statement

The studies involving human participants were reviewed and approved by Peking Union Medical College Institutional Review Board. Written informed consent to participate in this study was provided by the participants' legal guardian/next of kin. Written informed consent was obtained from the individual(s), and minor(s)' legal guardian/next of kin, for the publication of any potentially identifiable images or data included in this article.

## Author Contributions

LS and RW conducted Sanger sequencing, analysis of data, and manuscript writing. AK and RW clinically characterized patients, collected blood samples, and extracted DNA. HZ, SH, and XH assisted with the family analysis and PCR performance. XZ and RW designed the study and supervised the study progress. All authors read and approved the final manuscript.

## Conflict of Interest

The authors declare that the research was conducted in the absence of any commercial or financial relationships that could be construed as a potential conflict of interest.
